# Poisonous Properties of the Sulphate of Quinine

**Published:** 1847-05

**Authors:** 


*Poisonous properties of the Sulphate of Quinine.*—Doctor Baldwin, of Mongomery, Alabama, details in the last number of the *American Journal of the Medical Sciences*, several cases illustrative of the poisonous action of quinine under certain circumstances. One is as follows:

A man, about 30 years old, habits and constitution good has had a cold for five or six days. On the morning of the 25th October, 1843, was taken with a chill, the sensation of coldness continuing nearly all day with severe and acute pain in the right side, frequent cough and severe headache.

26th. Pulse 108, rather soft and moderately full; skin hot; cough frequent; expectoration scant and fluid, slightly tinged with blood; severe headache, and great turgidity of veins about forehead; tongue nearly natural; bowels rather costive; thirst; slight dullness on percussion over lower part of right lung, and nearly over the whole lung the natural respiratory murmur is replaced by an irregular undulatory sound, resembling somewhat that produced by the application of a 'sea-shell' to the ear. In the left lung the sounds are healthy. Eyes very much injected; slight delirium.—℞. Calomel grs.v; p. opii gr.$\frac{1}{4}$, M. Repeat every third hour. Mucilaginous drinks.

*Noon.* Pulse 115; has taken three pills; expectoration of a reddish orange; otherwise same. ℞. Antim. tart. grs.ii; aq. pura ℥iv, M. ℥ss every hour. ℥iv of blood from frontal vein.

*Vesp.* Pulse 120, full and soft; respiration 36; dullness extending upwards; no sound present over dull part except a slight friction sound, more audible during inspiration; over sonorous part of lung on applying stethoscope only the undulatory sound heard, respiratory murmur being absent; cough frequent and attended with great pain; expectoration same; tongue moist; collection of sordes on upper teeth; breath fœtid; quite delirious, talking incoherently, and is anxious to get up and leave the bed; complains much of head and side. Took four successive doses of medicine, but vomited up a large quantity of bile just before taking the last dose. ℞.—Continue antimonial every hour.

8 o'clock, P.M. Pulse 104; respiration 28; sweating very freely; says he feels easier, since he had a large, thin, yellow operation, about half an hour ago.

10 P.M. Sleeps apparently easy for a few minutes and then awakes and talks deliriously. Pulse 98, full and soft; respiration 26; tongue rather disposed to be dry on dorsum; skin moist; complains of sharp pains darting through head; side easier. ℞. Continue antimonial every third hour; quin. grs. xii at 11 o'clock, grs. viii at 2 o'clock, and grs. viii at 5 o'clock in the morning.

27th. Pulse 104, more firm; respiration 28; skin became dry at quarter after 10 o'clock last night, and so continues; continued to have short naps, between which he would be awake and delirious; had three thin dark evacuations—after the second he took fifteen drops tr. opii, same quantity after third. He missed one dose of quinine (8 grs.) last night; complains much of the same darting pains through head and side; took about 12 oz. blood, from which he experienced no immediate relief of pain; pulse softened a little under it, the frequency remaining the same; no nausea; a little deaf; suppressed cough. ℞. (Now 7 o'clock, take quin. grs.viii.) Antimonial mixture (¼gr.) every hour; tr. opii gtt. x after each evacuation.

*Noon.* Pulse 100, softer; respiration same; skin moist; one more evacuation; sleeps steadily now, except when he is awakened to take medicine. ℞. Continue same.

2 P.M. Asleep, and sleeps soundly nearly all the time except when awakened to take medicine; pulse 100, full; respiration 28; skin hot and dry; tongue moist and white; cough frequent; expectoration scant, opaque, and without blood; slightly deaf; nausea. ℞. Continue same.

*Vesp.* 5 P.M. Is scarcely at all delirious; forehead moist; thirst; complains much of weariness, emptiness and nausea; pulse 98; respiration 24; sleeps well; has been up several times, but no evacuation. ℞. Give quin. grs.viii; continue antimonial.

8 P.M. Pulse 98; respiration 26; otherwise same. ℞. Quin. grs.xxvi; calomel grs.xii; tart. emetic grs.iss; p. opii grs.i; M. In pls. No. xviii, div.; three every second hour. Continue antimonial, gum water, &c.

28th. Pulse 85; respiration 20; one evacuation; less dullness; expectoration easy and free, opaque and yellowish; tongue moist and white. ℞. Continue same, leaving tartar emetic out of pills, and instead ℞. antim. tart. grs.iii; water ℥iv. M. ℥ss every hour.

*Noon.* Pulse 98; respiration 26; skin hot; expectoration more difficult and scant; has just thrown up some bile; considerable nausea; an orange colored matter deposited on the teeth, at the junction between them and the gums. ℞. Continue same, omitting one dose of the antimonial solution.

3 P.M. Called to see him. He had a little while before been taken with a *jerking motion* of the whole body, which lasted several minutes, and immediately after his *vision was so imperfect* that he could scarcely distinguish anything.—When I arrived, whole surface hot; respiration irregular, from 11 to 20; pulse 100, full and rather firm; temporal veins turgid, and temporal arteries throbbing; *great restlessness*, anxiety, and alarm; thirst increased; tongue more dry, white and rough; *pupils dilated;* he dozes two or three minutes at a time, then starts up breathing more quickly and audibly; cough frequent and dry; respiratory murmur heard over greater extent of lung. The convulsive movements of body came on every ten or twelve minutes, sometimes apparently of whole body, at others confined to the arms; in the latter case, by grasping the arms with moderate firmness, or pressing them against the bed, the motions could be arrested immediately. He was not insensible during the convulsions, nor was there foaming at the mouth, but occasionally a staring and vacant look, and rolling up of the eyes. By half after 4 o'clock *he was completely blind.* On drawing (4½ o'clock) an ounce of blood, from a small orifice, it jetted out with force and was the color of arterial blood, and much buffed when cold. From 4½ o'clock till 6 o'clock he had but one slight convulsion. After taking 1 oz. of blood I thought the pulse softened, but regained its same character by the time I had tied the arm.

It is unnecessary to pursue the subsequent details of the case. The man recovered after a protracted and serious illness, and has regained a useful degree of vision. His vision began to improve about 24 hours after, though it is still impaired up to this time.

He thinks such accidents may be avoided by giving the medicine largely diluted:

When the stomach is in a condition to bear it, its absorption and activity can be greatly facilitated by *largely diluting* it with warm water. When dissolved and diluted in this way, even when given in what may be termed 'heroic doses,' provided the quantity to be taken is divided and given at intervals of one, two, or three hours, its deleterious effects may always be avoided *and the remedy persisted in with safety* in the absence of any manifestation of its influence, which we should never feel safe in doing when giving the 'insoluble di sulphate,' whether in the form of pills or powders; for, in the former case, we will always be advised of its unfavorable influence early enough for a timely withdrawal or modification of the dose. When given in this way, a given quantity seems to produce a greater effect and in a shorter time, than even a larger quantity when prescribed in either of the other forms.

This mode of giving quinine cannot be urged too strongly, for besides being a *saving* of a valuable and costly drug, and a preventive of its poisonous effects, it will be found of great assistance in prescribing generally. In cases requiring much delicacy and determination in their management, and where the propriety of the prescription is a matter of debate with the physician himself, and he wishes to be made sensible of the effect of the first dose of his remedy, in order to determine upon a continuance, it becomes a matter of great moment that the medicine should be administered in that form in which it will be most certainly and speedily appropriated by the system, and its operation made manifest to the physician.

